# Laser Photoacoustic Detection of CO_2_ in Old Disc Tree-Rings

**DOI:** 10.3390/s100403305

**Published:** 2010-04-04

**Authors:** Boris Ageev, Yurii Ponomarev, Valeria Sapozhnikova

**Affiliations:** Institute of Atmospheric Optics of Siberian Branch of the Russian Academy of Sciences, Academician Zuev Square, 1. Tomsk, 634055, Russia; E-Mails: ageev@asd.iao.ru (B.A.); yupon@iao.ru (Y.P.)

**Keywords:** laser photoacoustic gas analysis, carbon dioxide, tree-rings, H_2_O, PACS: 42.62.Fi

## Abstract

A homemade CO_2_–laser photoacoustic spectrometer has been used for monitoring CO_2_ in gas samples extracted under vacuum from the wood of old spruce disc tree-rings for a ∼60 year series. The experimental results show that (1) the CO_2_ concentration exhibits annual trends correlated with an increase in atmospheric CO_2_ in a number of cases; (2) at the time when the annual CO_2_ trend changes from positive to negative, the annual tree-ring stable carbon isotope ratios (δ^13^C) of CO_2_ change as well; (3) the disc tree-ring widths are observed to decrease in most cases where the annual CO_2_ increased; (4) simultaneously with the annual CO_2_ variation, annual H_2_O distribution was detected in gas samples of the wood tree-rings of one spruce disc. The observed patterns of the annual CO_2_ distribution in the disc tree-rings are assumed to be the evidence of the impact of the atmospheric CO_2_ increase. In other words, a change in the concentration gradient between the stem and the atmospheric CO_2_ may lead to a gradual CO_2_ accumulation in the stem because of a decrease in the diffusion rate and to a change in the tree parameters.

## 1. Introduction

It is recognized that due to a high CO_2_ concentration in the stem of a living tree [1], an increase in the atmospheric CO_2_ produce minor changes in the concentration gradient between the atmospheric and stem CO_2_. Thus an increase in the atmospheric CO_2_ does not significantly affect the diffusion of CO_2_ from the tree stem into the atmosphere. However, an annual change in the concentration gradient due to the increase in the atmospheric CO_2_ does exist (and will exist in the future) and we believe that the dynamics of this process must be evident in the CO_2_ concentration trend in the stem. We have hypothesized [2] that the wood of tree-rings of old tree discs where a large amount of CO_2_ has been preserved [3] may contain this information, since gases involved in metabolism of a living tree in each of the years studied are sorbed by a capillary-porous system of the ring wood and may be preserved for a long time within stems. In this work, we report the results of a laser photoacoustic gas analysis of CO_2_ present in gas samples extracted under vacuum from annual rings of 5 spruce discs for a ∼60 year series, as well as results of correlation analysis of data versus the concentration of atmospheric CO_2_. Nowadays photoacoustic spectrometers based on different lasers are unique tools for gas testing [4–7], medical diagnostic by breath [8,9], *etc*.

## 2. Experimental Section

The concentration of CO_2_ (extracted under vacuum) in the wood of tree-rings was measured on 5 spruce discs, collected 30 km away from Tomsk in 2004. The mean radius and height of the trees were ∼26 cm and ∼23 m, respectively, and the number of rings was ∼85. All discs were stored under laboratory conditions until 2009, so the wood can be considered to be room-dried. The measurements were made using a homemade laser photoacoustic spectrometer with a computer-controlled tunable waveguide CO_2_–laser [10]. The detection limit of the spectrometer for a laser power of 70 mW was 2 × 10^−5^ cm^−1^ and the measurement error was ±5%. Before the measurements, the system was calibrated using a CO_2_/N_2_ reference mixture. A maximum sensitivity of the photoacoustic detector was achieved for a total pressure of 100 Torr. All measurements were performed at this pressure. To prepare the samples for the analysis, the wood of 4 rings was planed down using special chisels. Then the samples were weighted (3–20 g) and placed in 4 sealed exposure chambers. The latter were evacuated down to a vacuum of ∼0.1 Torr for 1 min for gas diffusion stimulation. Because the larger the sample amount, the higher the gas pressure P(total) and P(CO_2_), it follows that P (CO_2_): P (total) [ppm] = const, irrespective of the sample weight. Then the exposure chambers were allowed to stay for 40 min to ensure a maximum yield of gases sorbed by the wood. Next, an exposure chamber was connected to the evacuated photoacoustic detector so that the gas samples enter the detector and air was admitted to get a total pressure of 100 Torr. We have analyzed each ring gas sample at the same pressure (∼6 Torr) and in the same volume (photoacoustic detector). The signal amplitudes *U*_m_ from the analyzed gas sample (100 + 6 Torr) and from air *U*_a_ (at 100 Torr) were recorded, and the difference Δ*U* = *U*_m_ – *U*_a_ was determined automatically. Using a calibration curve, we determined a relative CO_2_ content in the gas sample for each of 4 rings of the disc. Then, the wood of the next 4 rings was examined on CO_2_ and so on. The absorption by the gas samples in a detector of laser radiation at 3 laser lines 10P (20, 16, 14) was found to be approximately equal. For our purposes, use was made of an average value of absorption. The CO_2_ measurements were made at laser lines that did not coincide with water lines at a gas sample pressure of 6–8 Torr. The absorption coefficients of H_2_O in the P10 (20, 16, 14) laser lines calculated by the line-by-line method [11] with spectroscopic database HITRAN 2008 [12] were 10^2^–10^3^ times lower than the CO_2_ absorption coefficient (at 1 atm). We believe that these measurements refer mostly to CO_2_ in these lines, the water contribution is negligible small. The presence of water in the gas samples was detected from the signal in the CO_2_ laser line 10R (20) whose frequency coincided with the H_2_O absorption line center.

H_2_O detection experiments were carried out using a classical drying method [13]: milled wood of the rings was placed into weighting bottles and that were allowed to stay in a thermostat 100–103 °C for two days and the difference in weights was then determined.

The tree-ring stable carbon isotope ratio (δ^13^C) of CO_2_ chemically extracted from the wood rings was analyzed by an automated measuring MI-1201V Mass Spectrometer and DELTA V Advantage. The samples were prepared using standard certified methods of chemical preparation of organic substances. The results were reduced to the PDB international standard of Vienna Pee Dee belemnite for C.

## 3. Results and Discussion

In our earlier work [2], the annual CO_2_ distribution in the wood of tree-rings of old fir tree discs was reported for a 25-year series. It was found that in most cases, the CO_2_ content in gas samples extracted under vacuum from the annual rings of the discs was higher than the atmospheric CO_2_. Moreover, an annual trend of the CO_2_ concentration exists and in some cases, it correlates with atmospheric CO_2_ (and O_3_ [14]).

Measurements of CO_2_ extracted under vacuum from the wood of the tree rings of 5 spruce discs were performed for a ∼60 year series (1940–2004) using the homemade laser photoacoustic spectrometer.

### 3.1. Correlation between Tree-Rings and Atmospheric CO_2_

**Figure 1.**
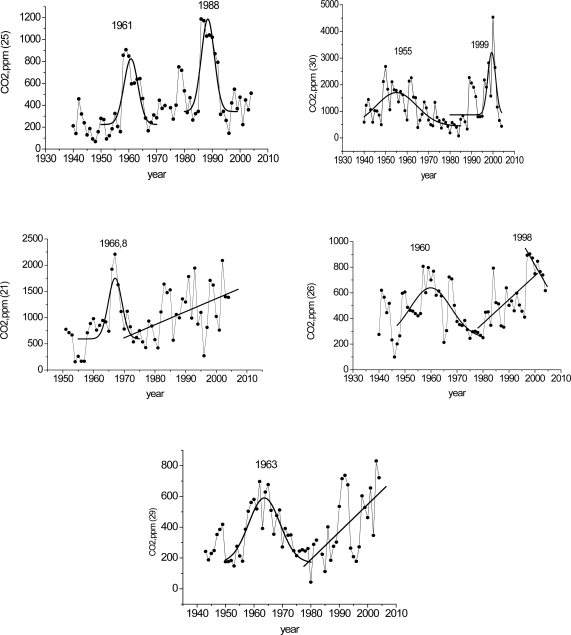
Annual trends of the CO_2_ concentration in spruce discs (No. 21, 25, 26, 29, 30) obtained with the use of a Gaussian approximation for identifying a maximum content.

The measurements of the CO_2_ content in the wood of the rings of 5 spruce discs revealed certain characteristic features: in all discs the first CO_2_ concentration maximum in the tree-rings was roughly dated to the 1960s, whereas the second maximum observed in certain discs was dated to ∼1988–1999 (Figure 1). Strong CO_2_ fluctuations were caused by structural heterogeneity of the biological objects under study and partly by the difference in wood ring destruction during sample preparation. However, even for such a spread in the results, the tendency for the CO_2_ growth in the discs is well pronounced.

We compared our CO_2_ measurements in the tree-rings with the atmospheric CO_2_. To this end, all annual CO_2_ trends were divided into annual sections and the years where CO_2_ increased were compared with the those of Mauna Loa data on an increase in the atmospheric CO_2_ (2008, http://cdiac.ornl.gov/ftp/trends/co2/maunaloa.co2). If a linear approximation could be used for our data and those of Mauna Loa, the correlation between them was determined using Origin software package and the correlation coefficients R and P values (probability that R is zero) were estimated.

The results of the correlation are presented in Table 1. Assuming the significance level of P ≤ 0.05, we may conclude that the observed positive CO_2_ trend in the disc tree rings for the above indicated CO_2_ fluctuations correlate well with the increase in the atmospheric CO_2._ The change of the sign of the trend seen in the 1990s was observed by a number of investigators who carried out isotope analyses for cellulose carbon in tree rings [15,16]. The decrease in the tree-ring δ^13^C was explained in the following way: “Tree-ring δ^13^C chronologies showing a decline over recent decades, that is not explained by a parallel change in the controlling climatic variables, have been reported from several areas, including the Swiss Alps, north-eastern France. McCarroll *et al.* (2009) suggest that this is threshold effect, with trees having reached the limits of physiological adaptation to increasing CO_2_ levels in the atmosphere” [17]. Conceivably, the change of the sign of the trend in the annual CO_2_ distribution (maximum) in the disc and cellulose carbon is due to general metabolic processes. We recorded another maximum as early as the 1960s. It is evident in Figure 1 that, for certain discs, the annual CO_2_ growth in the 1990s did not reach a maximum, probably due to tree growth conditions.

### 3.2. The Sign of the CO_2_ Trend Associated with a Change in Metabolic Processes

**Figure 2.**
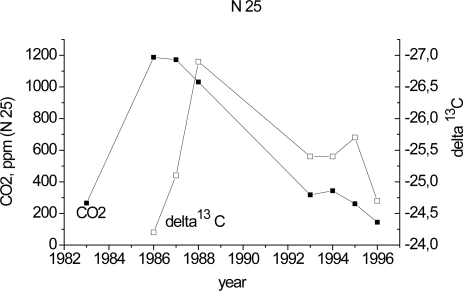
Annual trend of the CO_2_ concentration in a spruce disc (No. 25) and δ ^13^C isotope composition (delta^13^C).

According to our earlier assumption [18], the change of the sign of the trend (*i.e.*, a decrease in the CO_2_ concentration in the discs after its increase) is attributable to any change in metabolic processes. This assumption can be verified by analyzing the tree-ring stable carbon isotope ratios of CO_2_ (δ^13^C). It is known that terrestrial plants (C_3_ type, including conifers) are characterized by a range of isotope compositions (δ ^13^C) from approximately −22‰ to −32‰, while the isotope composition of the atmosphere is on average −7‰ [19]. The results obtained from an investigation of (δ ^13^C) in the time period where the disc CO_2_ (spruce 25) changes the sign are illustrated in Figure 2. It is obvious that CO_2_ was formed by the tree itself. Stable carbon isotope ratios (δ ^13^C) of CO_2_ changed as well, along with the annual CO_2_ variations in the tree-rings.

**Figure 3.**
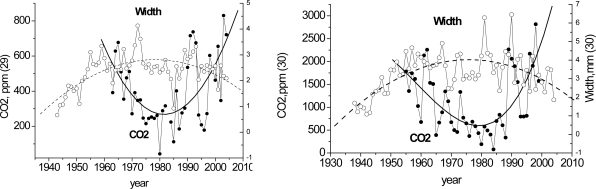
Annual trends of the tree-ring CO_2_ concentration and widths of spruce discs (No. 29, 30).

We compared our annual CO_2_ distribution over spruce disc tree-rings with the ring widths. In most cases, the annual ring width narrows with increase in the annual CO_2_ (Figure 3).

### 3.3. Presence of CO_2_ in Tree-Ring Water

**Figure 4.**
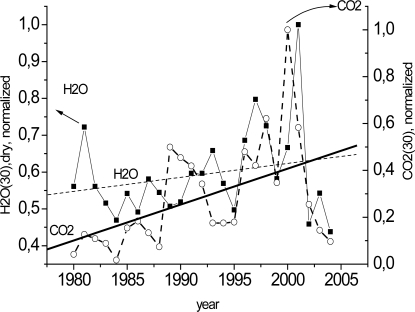
The tree-ring CO_2_ and H_2_O concentrations (normalized to unity) of a spruce disc (No. 30) obtained by two methods.

Early in the experiment, it was assumed that CO_2_ was sorbed only by the wood capillary-porous system. The results showed that tree-ring gas samples contained not only CO_2_ but H_2_O as well. Figure 4 illustrates the annual CO_2_ distribution (normalized to unity) measured by the photoacoustic method and the annual H_2_O distribution measured by the classical drying method in the tree-rings (disc 30). As seen from Figure 4, CO_2_ and H_2_O demonstrate nearly identical trends. This leads us to suggest that CO_2_ also exists in water solution. There are many papers concerning the moisture content in wood (see, e.g., [20,21]), but none of them considers annual H_2_O distribution in old disc tree-rings.

## 4. Conclusions

It is generally accepted that a long stay of tree discs under atmospheric conditions makes the stem-contained excess CO_2_ to decrease down to the atmospheric CO_2_ level due to diffusion processes. However, the results of our measurements of the CO_2_ extracted under the vacuum from the wood of tree-rings of old discs by the photoacoustic method have shown that (1) in most cases, the relative CO_2_ content in gas samples exceeds the atmospheric CO_2_ concentration; (2) certain annual trends of the CO_2_ concentration are observed; (3) the trend of the annual CO_2_ concentration in the rings of spruce discs changes the sign (from positive to negative) and the tree-ring stable carbon isotope ratios (δ ^13^C) change as well; and (4) in the time period where the CO_2_ concentration in the wood of tree-rings increases, the ring widths change (they are generally narrowed).

It is our opinion, that the observed CO_2_ trends are associated with the increase in the atmospheric CO_2_ content: the permanent growth of the atmospheric CO_2_ always decreases the CO_2_ diffusion rate from the stem, thus resulting in CO_2_ accumulation and, probably, in a change of certain internal metabolic processes. It is impossible to detect such a trend in a living tree, whereas the analysis of the wood sorbed gas can reveal its existence. We can also assume that the increased sorbed CO_2_ concentration in the tree-rings may be attributed to the ring narrowing, which conceivably explains why the trees do not show large radial increment when the temperature and atmospheric CO_2_ increase. The results obtained show that old discs contain valuable information that can be used for estimating the carbon budget in forests.

## Figures and Tables

**Table 1.** Correlation between spruce disc and atmospheric CO_2_.

Spruce No.	Total number of tree rings	Time period	Correlation coefficients R	Probability (that R=0) P	N, number of measurements (data points)
21	∼75	1970–2004	+0.5	0.00216	34
25	∼90	1966–1993	+0.66	1.9×10^−4^	27
26	∼80	1975–1997 1938–1960	+0.53 +0.73	0.01 0.01	22 11
29	∼80–90	1980–2004	+0.59	0.00261	24
30	∼150	1988–2000	+0.69	3.6×10^−4^	22

## References

[1] Teskey R.O., Saveyn A., Steppe K., McGuire M.A. (2008). Origin, fate and significance of CO_2_ in tree stems. New Phytol.

[2] Ageev B.G., Ponomarev Yu, N., Sapozhnikova V.A. (2009). Photoacoustic analysis of CO_2_ content in annual tree rings. J. Appl. Spectrosc.

[3] Ageev B.G., Nesvetailo V.D., Ponomarev Yu, N., Sapozhnikova V.A. (2005). Dendrochronoindication Using Gas Analysis. Sib. J. Ecol.

[4] Yun Y., Chen W., Wang Y., Pan C. (2008). Photoacoustic detection of dissolved gases in transformer oil. Eur. Trans. Electr. Power.

[5] Sigrist M.W. (2003). Trace gas monitoring by laser photoacoustic spectroscopy and related techniques. Rev. Sci. Instr.

[6] Schramm D.U, Sthel M.S., da Silva M.G., Carneiro L.O., Junior A.J.S., Souza A.P., Vargas H. (2003). Application of laser photoacoustic spectroscopy for the analysis of gas samples emitted by diesel engines. Infrared Phys. Technol.

[7] Koskinen V., Fonsen J., Kauppinen J., Kauppinen I (2006). Extremely sensitive trace gas analysis with modern photoacoustic spectroscopy. Vib. Spectrosc.

[8] McCurdy M.R., Bakhirkin Yu., Wysocki G., Lewicki R., Tittel F.K. (2007). Recent advances of laser-spectroscopy-based techniques for applications in breath analysis. J. Breath Res.

[9] Wang C., Sahay P. (2009). Breath analysis using laser spectroscopic techniques: breath biomarkers, spectral fingerprints, and detection limits. Sensors.

[10] Sherstov I.V., Bychkov K.V., Vasiliev V.A., Karapuzikov A.I., Spitsin V.V., Chernikov S.B. (2005). Twochannel CO_2_ laser system for heterodyne lidar. Atmos. Ocean. Opt.

[11] Mitsel A.A., Ptashnik I.V., Firsov K.M., Fomin B.A. (1995). Efficient technique for line-by-line calculating the transmittance of the absorbing atmosphere. Atmos. Ocean. Opt.

[12] Rothman L.S., Gordon I.E., Barbe A., Benner D.C., Bernath P.F., Birk M., Boudon V., Brown L.R., Compargue A., Champion J.-P., Chance K., Coudert L.N., Dana V., Devi V.M., Fally S., Flaud J.-M., Gamache R.R., Goldman A., Jacquemart D., Kleiner I., Lacome N., Lafferty W.J., Mandin J.-Y., Massie S.T., Mikhailenko S.N., Miller C.E., Moazzen-Ahmadi N., Naumenko O.V., Nikitin A.V., Orphal J., Perevalov V.I., Perrin A., Predoi-Cross A., Rinsland C.P., Rotger M., Šimecková M., Smith M.A.H., Sung K., Tashkun S.A., Tennyson J., Toth R.A., Vandaele A.C., Vander Auwera J. (2009). The HITRAN 2008 molecular spectroscopic database. J. Quant. Spectrosc. Radiat.

[13] Ermakov A.I., Arasimovitch V.V., Smirnova-Ikonnikova M.I., Yarosh N.P., Lukovnikova G.A. (1972). Methods for Biochemical studies of Plants.

[14] Zuev V.V, Ageev B.G., Bondarenko S.L., Savchuk D.A., Sapozhnikova V.A. (2006). Possibilities of use of gas composition of annual tree rings for bioindication of stratospheric ozone variations. Bioindication of stratospheric ozone.

[15] Bettger T. (2007). Tree rings as climate and environmental archives—stable isotope dendrological studies in Germany (Central Europe).

[16] Voronin V.I., Ivlev A.A. (2007). Dendroisotopical data indicates to modern chance of the climate of the earth. In.

[17] Sidorova O.V., Siegwolf R.T.W., Saurer M., Shaskin A.V., Knorre A.A., Prokushkin A.S., Vaganov E.A., Kirdyanov A.V. (2009). Do centennial tree-ring and stable isotope trends of *Larix gmelinii* (Rupr.) Rupr. indicate increasing water shortage in the Siberian north?. Oecologia.

[18] Ageev B.G., Ponomarev Yu, N., Sapozhnikova V.A. (2009). A trend of the CO_2_ concentration in tree rings and the atmospheric CO_2_. Atmos. Ocean. Opt.

[19] Galimov E. (1981). Nature of Isotope Biological Fractionation.

[20] Hartley I.D., Kamke F.A., Peemoeller H. (1992). Cluster theory for water sorption in wood. Wood Sci. Technol.

[21] Fromm J.H., Sautter I., Matthies D., Kremer J., Schumacher P., Ganter C. (2001). Xylem water content and wood density in spruce and oak trees detected by high-resolution computed tomography. Plant Physiol.

